# Patients’ Experiences of Outcome Expectations in Renal Denervation: A Qualitative Study

**DOI:** 10.3390/healthcare14172786

**Published:** 2026-09-01

**Authors:** Dandan Huang, Dayu Tang, Xi Chen, Shuting Zhu, Meiqi Yao, Jianping Song

**Affiliations:** 1 Department of Nursing, The Second Affiliated Hospital of Zhejiang University School of Medicine, Hangzhou 310009, China; huangdandan@zju.edu.cn (D.H.); 2508089@zju.edu.cn (X.C.); zhushut@zju.edu.cn (S.Z.); zrhlyj@zju.edu.cn (M.Y.); 2 School of Nursing, Zhejiang University School of Medicine, Hangzhou 310029, China; 22418900@zju.edu.cn

**Keywords:** renal denervation, uncontrolled hypertension, outcome expectations, qualitative study, nursing

## Abstract

**Highlights:**

**What are the main findings?**

- Patients’ outcome expectations regarding renal denervation are shaped by treatment experiences, perceived disease risks, medication burden, and social influences.
- Patients retrospectively described their outcome expectations as changing across preoperative decision-making, perioperative preparation, and postoperative adaptation, involving expectation formation, regulation, and readjustment.

**What are the implications of the main findings?**

- Nurses should address both cognitive and emotional dimensions of outcome expectations through structured assessment and evidence-based communication.
- Long-term follow-up and continuous professional support are essential for helping patients develop realistic expectations and adapt to postoperative outcomes.

**Abstract:**

**Objectives:** To investigate the outcome expectations of patients undergoing renal denervation (RDN), clarify the contextual factors contributing to their development, characterize their content and attributes, and provide evidence for the development of structured perioperative intervention strategies. **Methods:** Purposive sampling was used to recruit 17 patients undergoing RDN as interview participants. Guided by Social Cognitive Theory (SCT), a qualitative descriptive design was adopted, and semi-structured interviews were conducted. Data were analyzed using inductive thematic analysis. **Results:** Three major themes and 10 subthemes were identified: formation of outcome expectations (expectations triggered by prior treatment frustration, expectations strengthened by risk perception, treatment expectations driven by medication burden, and expectations shaped by social information); regulation of outcome expectations (cognitive trade-offs triggered by uncertainty regarding RDN efficacy, and establishment of defensive psychological expectations); and readjustment of outcome expectations (validation of expectations based on outcome feedback, emotional experiences triggered by expectation discrepancy, psychological adjustment driven by unfavorable outcomes, and behavioral adjustment guided by support needs). **Conclusions:** Outcome expectations among patients undergoing RDN for hypertension management are dynamic and complex. These findings suggest that multidimensional assessment, individualized information guidance, attention to cognitive and emotional expectations, and continuing follow-up support may be useful directions for future nursing research and practice. Such measures may support the development of realistic outcome expectations, reduce postoperative psychological discrepancies, and improve treatment adherence and satisfaction.

## 1. Introduction

According to the World Health Organization [1], the global population of patients with hypertension has exceeded 1.4 billion, yet effective blood pressure control is achieved in only approximately 25% of patients [2]. Poor long-term blood pressure control represents a major risk factor for cardiovascular and cerebrovascular events. Although various therapeutic modalities are available, effective blood pressure control remains difficult in a subset of patients. Although resistant hypertension has traditionally been regarded as an important indication for renal denervation (RDN), a considerable proportion of patients with uncontrolled hypertension continue to have difficulty achieving blood pressure control despite comprehensive management [3].

Renal denervation (RDN), an emerging minimally invasive interventional therapy, has been recommended by contemporary guidelines and expert consensus as a treatment option for appropriately selected patients with uncontrolled or resistant hypertension after comprehensive clinical evaluation [4,5,6]. Current decision-making for RDN is based on multiple factors, including blood pressure phenotype, optimization of antihypertensive therapy, medication adherence and tolerance, exclusion of secondary hypertension, patient preference, and shared decision-making, rather than resistant hypertension alone. However, its clinical efficacy varies across individuals, and postoperative blood pressure control may fail to meet expectations in some patients. In clinical practice, discrepancies are frequently reported between patients’ actual postoperative experiences and their preoperative expectations. Outcome expectations refer to individuals’ beliefs about the likely consequences of a particular behavior or course of action. In this study, they specifically refer to patients’ anticipated outcomes of undergoing RDN, including changes in blood pressure control, medication use, treatment burden, and quality of life [7]. Outcome expectations are a central construct within SCT, which further emphasizes reciprocal interactions among personal, behavioral, and environmental factors. Evidence indicates [8] that realistic and positive outcome expectations may promote patient engagement in disease self-management while improving postoperative coping capacity, treatment adherence, and physician–patient collaboration. Conversely, unrealistic or excessively high expectations may result in discordance between anticipated and actual treatment outcomes, adversely affecting postoperative satisfaction, emotional well-being, and long-term health behaviors, while increasing the likelihood of repeat interventions.

Current domestic and international research on RDN has primarily focused on its antihypertensive efficacy and safety [5,6], whereas limited attention has been directed toward the mechanisms through which patients’ outcome expectations are formed and subsequently adjusted during treatment decision-making and postoperative adaptation. This issue is especially important because RDN remains an emerging interventional technique with inherent uncertainty regarding therapeutic efficacy. Within this context, patients’ outcome expectations are highly dynamic and complex, warranting detailed investigation of their developmental pathways and influencing factors. SCT emphasizes reciprocal interactions among personal, behavioral, and environmental factors and identifies outcome expectations as an important cognitive determinant of health-related decision-making and behavioral regulation. In this study, outcome expectations constituted the primary theoretical construct, while self-efficacy, environmental influences, and behavioral regulation were used as sensitizing concepts to understand how these expectations were formed, regulated, and readjusted. Accordingly, SCT was considered appropriate because it enabled patients’ expectations regarding RDN to be interpreted in relation to their prior treatment experiences, information obtained from clinicians and peers, and subsequent self-management and support-seeking behaviors. Guided by SCT, this study used qualitative methods to examine the formation, regulation, and readjustment of outcome expectations among patients undergoing RDN, thereby offering preliminary insight into patients’ expectation-related experiences and providing an exploratory basis for outcome expectation management in perioperative and continuing nursing care.

## 2. Participants and Methods

### 2.1. Participants

**Figure 1:**
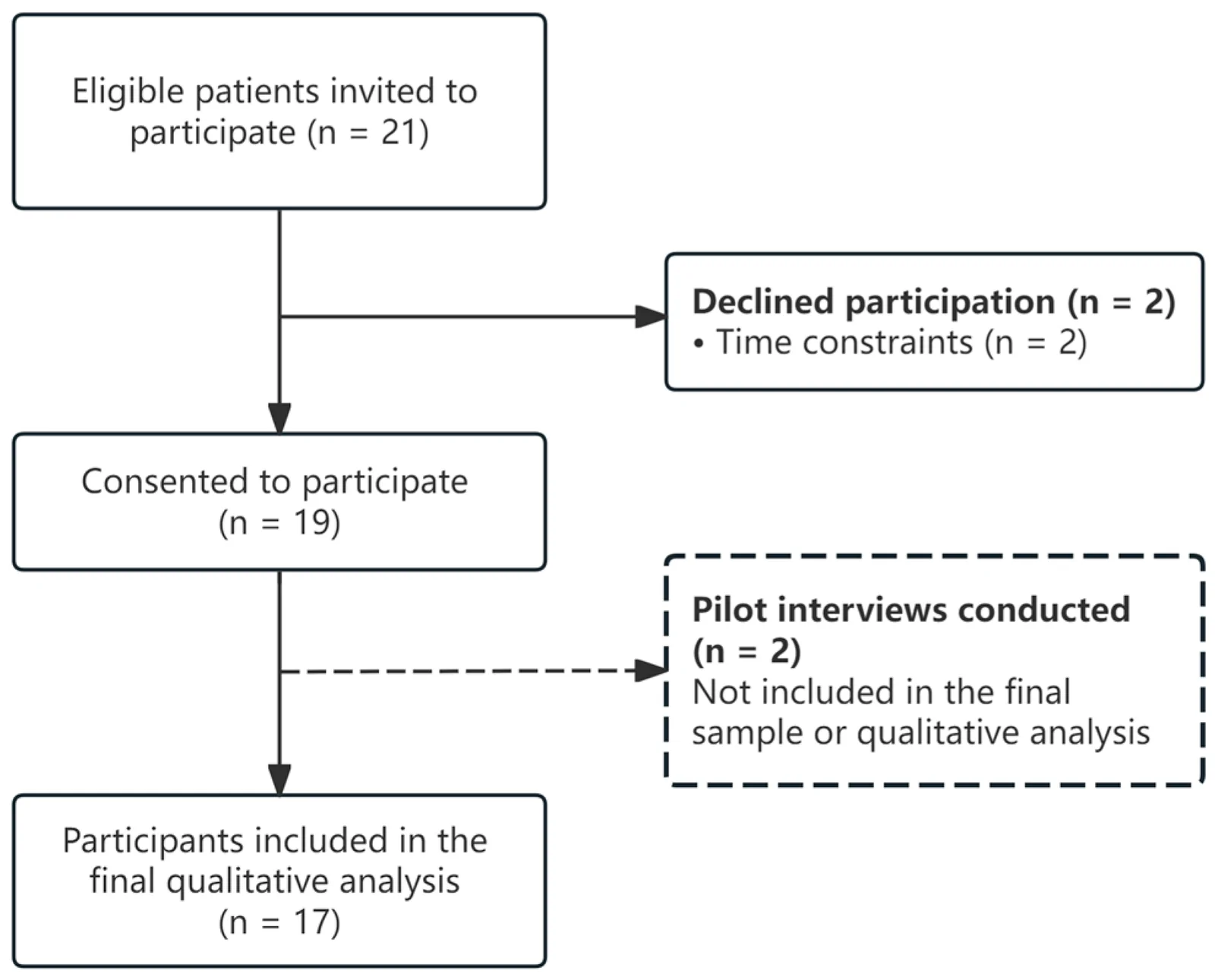
Flow diagram of participant recruitment and inclusion.

From January to March of 2026, purposive sampling incorporating maximum variation sampling was used to recruit patients undergoing RDN from the cardiology departments of different campuses of our hospital. To capture diverse experiences related to RDN decision-making and outcome expectations, maximum variation sampling was guided by predefined demographic and clinical characteristics, including age, sex, duration of hypertension, educational level, place of residence, type of medical insurance, number of comorbidities, and clinical characteristics related to hypertension management. Eligible patients were identified and recommended by the clinical team according to the study criteria, and the researcher subsequently contacted potential participants, explained the study objectives, interview procedures, confidentiality principles, and voluntary participation, and invited them to participate. The inclusion criteria were as follows: (1) patients undergoing RDN in accordance with the indications specified in the Chinese Expert Consensus on the Clinical Pathway for Percutaneous Renal Denervation in the Treatment of Hypertension (2025 Edition). The decision to perform RDN was based on comprehensive clinical assessment rather than a diagnosis of resistant hypertension alone, including blood pressure phenotype, optimization of antihypertensive therapy, medication tolerance, exclusion of secondary hypertension, patient preference, and shared decision-making [9]; (2) age ≥ 18 years; and (3) adequate verbal expression and communication ability. The participants represented patients undergoing RDN in routine clinical practice rather than exclusively individuals with confirmed true resistant hypertension. The exclusion criteria were as follows: (1) current participation in other intervention programs; and (2) severe cardiac or renal insufficiency, an acute medical event, or cognitive impairment that could impede participation in the interview. The sample size was guided by thematic saturation, defined as the point at which no new subthemes or themes emerged during concurrent data analysis. Saturation was jointly assessed by two researchers (D.T. and S.Z.), and two further interviews were conducted to confirm this assessment. During the recruitment period, 21 eligible patients were invited to participate; 2 declined because of time constraints, and 19 consented to participate. Of these, 2 participated in pilot interviews and were not included in the final sample or qualitative analysis, leaving 17 participants in the final qualitative analysis (Figure 1). Their characteristics are presented in Table 1 and Table 2. This study was approved by the Institutional Review Board of the Second Affiliated Hospital, Zhejiang University School of Medicine (Approval No.: [2026] Ethical Review Study No. [0123]).

### 2.2. Methods

#### 2.2.1. Development of Interview Guides

On the basis of the study objectives and relevant literature, a preliminary interview guide was developed with outcome expectations as the primary construct and self-efficacy, environmental influences, and behavioral regulation as sensitizing concepts for exploring the formation, regulation, and readjustment of patients’ expectations. The draft was subsequently refined through discussion and revision by the research team. Two patients were invited to participate in pilot interviews to further assess the clarity, comprehensibility, and appropriateness of the interview guide. The two pilot interviews were not included in the final sample or qualitative analysis. Based on feedback from the pilot interviews, minor revisions were made to improve the wording and sequence of the interview questions before formal data collection. The final interview guide included the following questions: (1) Could you describe your illness and treatment experiences before deciding to undergo RDN? (2) In your opinion, what are the main reasons for the suboptimal long-term control of your blood pressure? (3) What specific reasons or expectations motivated your decision to undergo the RDN procedure? (4) Do you think the RDN procedure has met your expectations? (5) In which specific aspects do you think this procedure may fail to meet your expectations? (6) What efforts have been made, or are planned, to better achieve your desired treatment outcomes? To what extent do you think these efforts can be sustained over the long term? (7) What types of assistance and support would you like to receive during your treatment process?

#### 2.2.2. Data Collection Methods

Data were collected through semi-structured telephone interviews. Telephone interviews reduced time and location constraints and may have facilitated participants’ open expression of their views. Researchers trained in qualitative research methods selected participants according to the predefined inclusion and exclusion criteria. The interviews were conducted by a senior cardiovascular nurse trained in qualitative research. The interviews were conducted after discharge, and the interviewer was not directly involved in participants’ RDN decision-making or postoperative treatment management. Patients were contacted by telephone, and the study purpose, content, voluntary nature of participation, confidentiality, and audio-recording procedures were explained in detail before informed consent was obtained.

All interviews were conducted by telephone after discharge, at varying intervals following RDN. With participants’ permission, each interview was audio-recorded in full. Probing, repetition, and reflective responses were used to encourage participants to describe their prior experiences and feelings in depth. The sequence of questions in the interview guide was flexibly adjusted according to participants’ real-time responses, and follow-up questions were used to obtain more comprehensive information while leading questions and value judgments were avoided. Each interview lasted 30–40 min. Before the interview was concluded, the core content was summarized and verified with the participant, allowing additional details or corrections to be provided.

All interviews were conducted in Mandarin Chinese and were audio-recorded with participants’ permission. The recordings were transcribed verbatim in Chinese, and the original transcripts were used during the coding and interpretation process to preserve the contextual meanings of participants’ experiences. For reporting purposes, representative quotations were translated into English by the first author and reviewed by a bilingual researcher with qualitative research experience. The research team compared the translated quotations with the original Chinese transcripts to ensure semantic accuracy and cultural appropriateness. Any discrepancies were discussed and resolved through consensus. The translated quotations were minimally edited for readability while maintaining the original meanings of participants’ expressions.

#### 2.2.3. Data Analysis

Consistent with the qualitative descriptive design of this study, interview data were analyzed using inductive thematic analysis [10,11,12]. Within 24 h after each interview, one researcher transcribed the recording verbatim and documented audible paralinguistic cues, including pauses, tone of voice, and emotional expressions, while a second researcher checked the transcript against the recording. The transcripts were returned to participants for verification. Fifteen participants confirmed the accuracy of the transcripts. The remaining two participants did not respond after two contact attempts. Only minor wording clarifications were made, and no codes or themes were modified as a result of transcript verification. Interview data were coded chronologically, and a separate file was established for each participant.

Two researchers repeatedly read the transcripts to become familiar with the data and independently conducted open coding. Meaningful units related to participants’ expectations, perceptions, emotions, and support needs were identified. Similar codes were then compared, merged, and grouped into preliminary categories. The research team reviewed these categories in relation to the study aim and refined them into subthemes and themes. Initial coding was conducted inductively without applying predetermined categories from SCT. After preliminary codes and categories had been developed, the research team used SCT as a sensitizing framework to interpret anticipated treatment consequences as outcome expectations, patients’ confidence in sustaining self-management behaviors as self-efficacy, information and experiences obtained from clinicians, family members, and peers as environmental influences and observational learning, and patients’ self-management and support-seeking responses as behavioral regulation. Disagreements in coding and theme interpretation were discussed until consensus was reached. NVivo 12.0 (Lumivero, Denver, CO, USA) was used for data management and coding. An audit trail documenting coding decisions and theme development was maintained throughout the analysis. The code-to-theme pathway and the completed COREQ (Consolidated Criteria for Reporting Qualitative Research) checklist [13] are provided in Supplementary Materials S1 and S2.

## 3. Results

**Figure 2:**
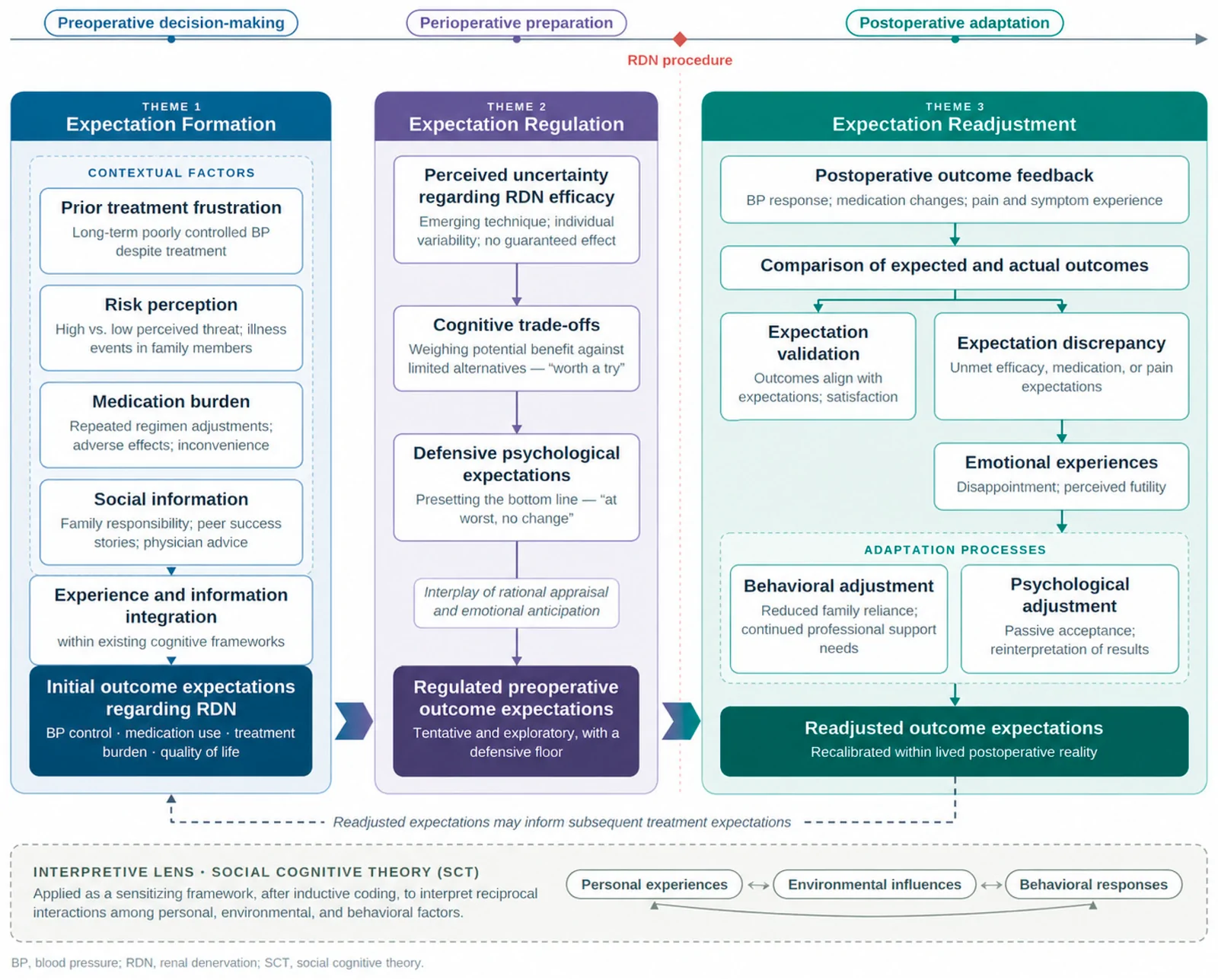
Conceptual framework of the formation, regulation, and readjustment of outcome expectations. **Note.** BP, blood pressure; RDN, renal denervation; SCT, Social Cognitive Theory. The dashed feedback pathway represents an interpretive relationship derived from participants’ retrospective accounts rather than a prospectively validated causal pathway.

Three themes and 10 subthemes were identified. The relationships among these themes and subthemes are summarized in a preliminary conceptual framework illustrating the formation, regulation, and readjustment of outcome expectations across preoperative decision-making, perioperative preparation, and postoperative adaptation (Figure 2).

### 3.1. Theme 1: Formation of Outcome Expectations

#### 3.1.1. Expectations Triggered by Prior Treatment Frustration

A history of poorly controlled blood pressure constituted the practical basis for patients’ expectations regarding RDN outcomes. Many participants described a long history of hypertension, and blood pressure remained chronically unstable, with recurrent elevations and reductions that were persistently difficult to stabilize. P13: “I first discovered it back in 2009; that makes it 16 years now.” Some participants reported persistent poor blood pressure control despite adhering to prescribed treatment regimens. Repeated experiences of uncontrolled blood pressure gradually contributed to a generalized perception that the condition was “uncontrollable” or “impossible to bring down.” P5: “The main issue is that no matter what medication I take, my blood pressure just won’t come down; the antihypertensive effect is poor.” P14: “My blood pressure medication regimen has been increased to six pills in the morning and four at night, yet for my hypertension, taking any amount of medication seems useless.” P7: “Over the past few years, I’ve consulted doctors at various hospitals and research institutes in Shanghai; sometimes, medication alone simply isn’t enough to keep it under control.”

#### 3.1.2. Expectations Strengthened by Risk Perception

Patients demonstrated varying perceptions of the long-term risks associated with hypertension, mainly reflected as either high risk perception, characterized by marked anxiety, or low risk perception, characterized by the belief that the condition was “not harmful at present.” Both extremes influenced patients’ outcome expectations regarding RDN. Some patients feared severe consequences, such as myocardial infarction, cerebral infarction, or sudden cardiac death, and adverse health outcomes among family members further heightened their perceived threat. As a result, RDN was more likely to be regarded as an important approach to risk reduction, and higher expectations were held regarding the efficacy of RDN. P1: “I am terrified that I might suddenly suffer a cardiac arrest or a cardiovascular blockage.” P2: “Hypertension can lead to problems like myocardial infarction, strokes, and atherosclerosis; I am very worried about my physical health.” Such fears were often closely associated with adverse health outcomes among family members, which further intensified patients’ perceived threat of disease-related consequences. P17: “My father also had hypertension; he passed away at the age of 52 due to a cerebral infarction.” P14: “I’m still very frightened because my father, my grandfather, and my aunt all suffered strokes after developing hypertension; my aunt is now paralyzed.”

Conversely, some patients tended to underestimate long-term risks and attempted to reduce the perceived threat of the disease by reasoning that it was “not affecting them at present.” Although treatment expectations were still present, RDN was often approached with a tentative attitude, and expectations regarding its therapeutic efficacy remained reserved. P9: “I don’t really mind either way; as long as it’s not affecting me right now, that’s fine—I’ll just deal with it when the time comes.” P16: “I’m a very easygoing person, so there isn’t really anything that gets to me.”

#### 3.1.3. Treatment Expectations Driven by Medication Burden

Patients’ experiences with pharmacological treatment represented an important driver in shaping their outcome expectations regarding the RDN procedure. Most patients had undergone repeated adjustments to antihypertensive medication regimens but still had difficulty achieving stable blood pressure control. P4: “The local hospital switched my medication three times, but (my blood pressure) still wouldn’t come down.” P9: “At the time, they kept adding more medications—up to four different drugs—but (the results) still weren’t effective.” P16: “I’ve been taking medication continuously, but the results haven’t been great; I started switching back and forth between different drugs, yet my blood pressure remained very high.” In addition, some patients had long been affected by medication-related adverse effects, which further diminished their tolerance of, and confidence in, existing treatment approaches. P15: “I tend to be quite sensitive (to medications); sometimes, after taking them, I experience coughing and swelling.” P3: “Taking this particular medication triggers eczema flare-ups for me. Topical creams offer temporary relief, but the moment I stop the medication, it recurs. It’s a constant cycle—it just won’t go away. Having eczema all over my body also makes my insomnia much worse.” Moreover, the inconvenience associated with long-term medication use had become a substantial burden for patients. P1: “I just feel that taking medication is such a hassle.” P3: “For instance, when traveling, I have to constantly worry about whether I’ve packed my medication. I also need to pay attention to the local temperature. If it differs by about ten degrees from that in my hometown, I will worry whether my regular dosage is still appropriate. It is really quite troublesome, and sometimes I even forget to take my medicine.” P11: “I’m supposed to take medication every day. Initially, I was very diligent about taking it daily, but as time went on, I started occasionally forgetting—missing a dose here or there—leading to an irregular medication schedule.” These experiences reflected the heterogeneous clinical backgrounds of patients undergoing RDN, including medication burden, adverse drug reactions, and challenges in maintaining long-term pharmacological treatment. Participants’ expectations regarding medication reduction varied considerably. While some patients viewed reduced medication use as an important indicator of treatment success, these expectations were related to perceived treatment burden and did not necessarily correspond to the clinical goal of medication discontinuation after RDN.

#### 3.1.4. Expectations Shaped by Social Information

On the basis of their existing cognitive frameworks, patients’ outcome expectations were further shaped by multiple external factors. Motivated by a sense of family responsibility, some patients were particularly inclined to pursue active treatment to prevent disease progression and avoid becoming a burden to their families. P5: “I’m terrified that if I fail to keep my blood pressure well controlled and end up having a cerebral hemorrhage, it will affect my whole family. My children and husband would have to put aside everything to look after me.” In addition, reports of successful outcomes among fellow patients or friends strengthened patients’ confidence in RDN efficacy, making positive postoperative recovery easier to envision and anticipate. P6: “I have a fellow patient whose blood pressure often soared above 200 mmHg. After the procedure, his blood pressure has been kept in good condition and remains extremely stable. It really shows that this procedure is highly effective.” P3: “A friend of mine also underwent this procedure. Now, he only needs to take a single medication, and his blood pressure remains perfectly under control.” Meanwhile, physicians, as medical professionals and authoritative experts, exerted substantial influence on patients’ treatment decisions. P16: “My blood pressure just wouldn’t come down; the doctor told me that procedure might be able to bring it down to a normal level.”

### 3.2. Theme 2: Regulation of Outcome Expectations

#### 3.2.1. Cognitive Trade-Offs Triggered by Uncertainty Regarding Procedural Efficacy

Before the procedure, most patients had acquired a preliminary understanding, either through communication with medical staff or independent information seeking, that RDN was not “100% effective.” This psychological preparedness was derived from preoperative discussions with physicians, as well as acceptance of individual variability in treatment outcomes and the inherent limitations of the procedural mechanism. P3: “My attending physician didn’t know (what results to expect) either; in fact, I only found out later that the specific ablation sites are chosen somewhat by chance.” P6: “Before the procedure, I didn’t really have any specific expectations regarding the outcome; it was a new technology, after all, so there were no guarantees.” P17: “The doctor told me at the time that (the surgical outcome) varies from person to person: for some, blood pressure can be controlled with slightly less medication, while for others, it remains largely the same as before.”

#### 3.2.2. Establishment of Defensive Psychological Expectations

When antihypertensive medication regimens reached their maximum tolerance yet blood pressure remained uncontrolled, most patients regarded RDN as an attempt to break through the therapeutic dilemma. They relieved decision-making pressure by presetting the bottom line that the worst outcome would be no change at all, showing an exploratory and tentative expectation for RDN efficacy. P5: “I never worried too much and was mentally prepared. It does not matter even if the procedure fails; at worst, everything will stay the same as before.” P6: “I kept wondering whether the worst result could be worse than my current condition. The worst case is simply no obvious effect, with blood pressure dropping only three to five mmHg. I could accept that, and I had to give it a try anyway.” P14: “I still want to have a try even if it may not work. It is necessary to explore all possible treatment options.” P8: “I just take it as an extra surgical attempt. There is a chance it might work. The worst outcome is merely no therapeutic effect at all.”

### 3.3. Theme 3: Readjustment of Outcome Expectations

#### 3.3.1. Validation of Expectations Based on Outcome Feedback

Postoperatively, patients generally reported high satisfaction. Most patients indicated that procedure had produced a substantial positive effect, resulting in effective blood pressure control and improved quality of life. Some patients perceived that procedure had successfully achieved their intended goals and reduced their dependence on medication. P5: “My blood pressure has finally come down and stabilized; my diastolic pressure isn’t quite so high anymore. The results are actually quite ideal.” P7: “I feel this procedure was definitely worth it. At the very least, it means I can take less medication.” P17: “I think the results are excellent; looking at the data, I am satisfied.” P16: “I do believe the procedure was effective; there has been an improvement compared to before, and it has largely met the expectations I had at the time.” P12: “Now, looking back, I feel it was well worth it, and it is consistent with what I expected.”

#### 3.3.2. Emotional Experiences Triggered by Expectation Discrepancy

Although many patients expressed satisfaction with postoperative outcomes, a considerable number perceived the results as falling short of their expectations, particularly because the procedure did not fulfill their preoperative outcome expectations, leading to evident disappointment. P15: “The first day (after the procedure) was fine, but then my blood pressure started rising again. It felt as though the procedure had been completely in vain, which left me feeling quite disappointed.” P2: “The results weren’t quite what I had imagined. I thought I would no longer need to take medication, but it turns out I still have to keep taking it.” Postoperative pain exceeding expectations also represented a major source of disappointment. P14: “It did not meet my expectations at all. I feel like the procedure was a total waste. The financial cost is one thing, and the pain I endured on the night after the operation was truly agonizing and almost unbearable.” P1: “The night of the procedure, my lower back pain was excruciating; I had to take painkillers just to find even slight relief.” Some patients acknowledged that procedure had produced certain beneficial effects, yet still perceived discordance between actual outcomes and their expectations. P13: “The treatment worked to some extent. I no longer suffer from dangerously high blood pressure, but the effect still cannot fully meet my preoperative expectations.” P4: “A gap remains between the actual results and what I expected; although the procedure certainly had some positive effect, the outcome simply isn’t quite as good as I had originally anticipated.”

#### 3.3.3. Psychological Adjustment Driven by Unfavorable Outcomes

After an initial period of disappointment, most patients gradually transitioned toward acceptance and adjustment to their actual outcomes. This acceptance was not derived solely from satisfaction with RDN outcomes; rather, in the context of limited superior alternatives, it represented a process of passive integration and psychological assimilation of the existing outcome. P14: “Yeah, so what can be done? This was my own decision; I can’t really blame the doctors.” P15: “There’s no other way; whether I like it or not, I just have to accept it.” P3: “Eventually, I came to terms with it and felt a sense of calm acceptance. I feel that things like this might just be fated—there’s nothing to be done about it.” This process indicated that patients’ outcome expectations did not end after the procedure; instead, they were reinterpreted and recalibrated within the lived reality of postoperative outcomes.

#### 3.3.4. Behavioral Adjustment Guided by Support Needs

During the management of outcome expectations, patients described a pattern characterized by reduced reliance on family support and continued needs for professional medical support. Specifically, blood pressure management tended to be regarded as a personal responsibility, with patients seeking to avoid imposing additional burdens on their families, while continued professional medical support was sought to meet expectations for continuous blood pressure feedback, medication guidance, and access to advanced information. P14: “I don’t need support from my family; I don’t want to trouble them.” P15: “Neither of us is very good at using smartphones… As for other forms of help (from family), I can’t really think of any, and it’s quite difficult for them to assist.” P6: “Regarding the safe limits for physical activity—at what blood pressure level is it safe to go swimming? Also, regarding intimate relationships within marriage, I worry about my blood pressure rising, so I really need guidance from a doctor. I can find some information online, but it’s not the same as professional advice.” P7: “If any new medications or technologies become available in the future, I hope you can let us know. For instance, if there are new techniques for treating hypertension that eliminate the need for long-term daily medication, or perhaps more convenient options like an injection that provides long-lasting control.” P9: “If there happens to be a new antihypertensive drug that is particularly effective, just recommend it to me so I can give it a try.”

## 4. Discussion

### 4.1. Strengthening Multidimensional Assessment and Information Guidance to Promote Realistic Patient Expectations

The study results indicated that outcome expectations among patients undergoing RDN were gradually shaped by the interaction of prior treatment experiences, risk perceptions, and information derived from environmental support. After prolonged poorly controlled blood pressure, repeated medication adjustments, and the burden of long-term pharmacotherapy, patients gradually develop the perception that conventional treatments have limited efficacy; this perception subsequently gives rise to expectations that RDN will improve their current condition. Meanwhile, patients’ risk perceptions regarding long-term complications, such as cardiovascular and cerebrovascular events, particularly when intensified by the projection of adverse health outcomes observed among family members, may increase their perceived disease threat and thereby elevate expectations regarding RDN efficacy. In addition, social information, including experiences shared by fellow patients and recommendations from physicians, continuously reinforces the formation of outcome expectations, making some patients susceptible to inflated or oversimplified expectations regarding treatment outcomes. The results are consistent with conclusions from previous studies on treatment decision-making behaviors among patients with chronic diseases, indicating that prior experiences of treatment failure and perceived disease-related risks are important drivers prompting patients to seek new therapeutic modalities [14,15]. This study further shows that, in the context of uncertainty regarding treatment efficacy, social information continuously influences the shaping of outcome expectations. This phenomenon reflects the effect of environmental factors on individual cognition and behavioral decision-making, as proposed by SCT [16]. Therefore, before RDN, healthcare professionals may consider assessing patients’ previous treatment histories and risk perceptions. Particular attention should be directed toward identifying “catastrophizing” interpretations, often triggered by disease-related events in family members, as well as inflated expectations. Structured health education and supportive communication strategies, such as motivational interviewing, may help patients develop more realistic outcome expectations, but these approaches require further testing in future studies [17,18].

The present findings also reflected the clinical heterogeneity of patients undergoing RDN. Rather than representing only patients with confirmed resistant hypertension, some participants reported medication intolerance, treatment burden, or difficulties maintaining long-term medication adherence. This observation is consistent with contemporary RDN practice, in which patient selection is based on comprehensive clinical evaluation rather than resistant hypertension alone.

Our findings are consistent with previous qualitative research showing that patients’ treatment expectations are shaped not only by symptom burden but also by previous treatment experiences, medication burden, interactions with healthcare professionals, and information obtained from family members and peers. Rather than being static, expectations develop gradually through continuous interpretation of disease experiences and healthcare encounters. Similar observations have been reported in studies of patient expectations and shared decision-making [19,20]. Recent qualitative research involving patients with severe difficult-to-treat hypertension has similarly highlighted the influence of treatment experiences and treatment goals on patients’ perceptions of RDN [21]. Qualitative research on new clinical procedures has also emphasized the importance of patient expectations, informed decision-making, and post-procedure follow-up in the context of uncertainty regarding risks and benefits [22]. The present findings extend this literature by showing that discrepancies between anticipated and experienced outcomes may contribute to disappointment and subsequent expectation readjustment after RDN.

### 4.2. Prioritizing Dual Assessment of Cognition and Emotion to Effectively Regulate Outcome Expectations

This study indicated that, before undergoing RDN, patients generally recognized that RDN efficacy varied across individuals and had established a certain degree of “defensive” psychological preparedness; nevertheless, they remained vulnerable to psychological disappointment after the procedure. This suggests that regulation of patients’ outcome expectations is not merely a rational cognitive process but rather a dynamic interaction between rational appraisal and emotional anticipation. From the perspective of SCT, decisions regarding health behaviors are not based solely on rational judgments about potential outcomes; they are also continuously shaped by prior experiences and emotional states. Although patients may cognitively accept the inherent uncertainty of treatment efficacy, heightened internal expectations may still be maintained, driven by previous treatment frustration and a strong desire for health improvement. When actual treatment outcomes fail to meet such expectations, even when objective improvement has been achieved to some extent, patients remain prone to disappointment and negative emotions. In addition, selective presentation of treatment outcomes on social media platforms, together with peer comparisons among patients, may further intensify postoperative discrepancies between expectations and reality [23]. Therefore, future expectation-support strategies may need to address not only patients’ rational understanding of treatment efficacy but also their emotional expectations. For informational interventions, individualized visual efficacy data, such as the degree of blood pressure reduction or target attainment rates, should be provided according to each patient’s specific circumstances. Contextualized explanations should also be used to help patients understand variability in treatment outcomes, thereby reducing unrealistic expectations. Meanwhile, the introduction of comparable cases for illustration may prevent excessive amplification of expectations based solely on isolated successful cases. This approach can guide patients toward outcome expectations consistent with clinical reality, thereby reducing posttreatment psychological discrepancies and improving follow-up adherence and treatment satisfaction [24]. A key contribution of this study is that it contextualizes patients’ subjective evaluations of RDN alongside their clinical blood pressure and medication profiles, showing that perceived treatment success depends not only on physiological improvement but also on whether outcomes align with individual expectations. Medication burden emerged as an important factor influencing patients’ expectations. However, medication burden should be distinguished from expectations that RDN will eliminate the need for antihypertensive therapy. Although some patients hoped to reduce or discontinue medications after RDN, current evidence and clinical counseling emphasize that medication reduction is not guaranteed and that antihypertensive therapy may still be required after the procedure. Therefore, preprocedural education should address realistic treatment goals, clarify the potential but uncertain changes in medication use, and help patients develop appropriate outcome expectations.

### 4.3. Strengthening Continuous Support and Follow-Up Management to Promote Readjustment of Outcome Expectations

This study showed that, after receiving postoperative “outcome feedback,” patients undergoing RDN demonstrated continuous and dynamic adjustment of their outcome expectations. When actual therapeutic outcomes did not fully align with expectations, patients often engaged in “psychological adjustment,” typically through strategies such as “passive acceptance” or “reinterpretation of results.” This process reflected the active reconstruction of outcome expectations under feedback mechanisms. Moreover, patients exhibited postoperative behavioral adjustment driven by support needs: on the one hand, blood pressure management tended to be regarded as a personal responsibility, with patients seeking to avoid burdening their families; on the other hand, a high level of dependence on professional medical support was observed, with expectations for continuous guidance and informational assistance. This duality may be attributable to patients’ internalization of hypertension management as a personal duty, which may, to some extent, inhibit emotional expression and proactive help-seeking behaviors. However, for blood pressure monitoring, dietary control, and exercise management, long-term adherence is difficult to sustain through individual willpower alone, suggesting insufficient continuity in the current support system. Therefore, a “family–medical” collaborative support model may be a potential direction for future intervention development. First, nursing staff should assess the specific types of family support available and distinguish emotional support from behavioral support functions. Family members should be guided to provide primarily nontechnical support, such as medication reminders, encouragement of blood pressure recording, and companionship during health-promoting activities, to enhance patients’ adherence to long-term management regimens [25]. Second, a structured follow-up pathway should be implemented, such as standardized follow-up time points [26]. This pathway should incorporate home blood pressure monitoring data for dynamic assessment, allowing timely and precise guidance on medication adjustment and lifestyle modification when blood pressure fluctuations occur. In addition, digital information platforms should be used for remote follow-up and the regular dissemination of medication adjustment recommendations and updates on advanced treatment options, thereby improving informational continuity and feedback timeliness [27].

Compared with previous qualitative studies focusing primarily on patient expectations or shared decision-making, the present study further demonstrates that outcome expectations surrounding RDN evolve dynamically through expectation formation, regulation, and readjustment. These findings extend current qualitative evidence by highlighting expectation management as a continuous process throughout the peri-procedural period rather than a single episode of preprocedural education.

### 4.4. Limitations

There are several limitations to this study. First, although participants were recruited from the cardiology departments of different campuses within the same hospital, this was a single-institution qualitative study with a relatively small sample size, which may limit the transferability of the findings. Although maximum variation sampling was applied, the sample included only two female participants, and some potentially relevant patient groups may have been underrepresented. Because eligible participants were identified with assistance from the clinical team and were interviewed about care received at the treating institution, selection bias and socially desirable responses, including possible reluctance to express criticism of the institution, cannot be excluded. Future multicenter studies with larger and more diverse samples are needed to further explore variations in outcome expectations among patients undergoing RDN. All interviews were conducted by telephone after discharge, and the interval between RDN and interview varied among participants. In addition, ambulatory blood pressure monitoring data were unavailable for all participants and were therefore not reported, which limited our ability to relate patients’ narratives to standardized out-of-office blood pressure outcomes. Therefore, participants’ accounts of preoperative expectations and perioperative experiences may have been influenced by postoperative experiences and recall bias, and the findings should not be interpreted as prospective longitudinal evidence. In addition, telephone interviews may have limited the researchers’ ability to capture nonverbal visual cues and contextual information. Although the interview guide was designed to use open-ended questions as much as possible, the wording of some questions during the interviews may have introduced a degree of interviewer or question-framing bias. Future studies should collect data at multiple time points before and after RDN and incorporate quantitative clinical outcomes to further examine changes in patients’ outcome expectations.

## 5. Conclusions

Guided by SCT, this qualitative study explored how patients retrospectively described the formation, regulation, and readjustment of outcome expectations before and after RDN. The findings provide a preliminary conceptual account of these interconnected experiences and may inform future prospective research and the development of expectation-support interventions. Potential directions include multidimensional assessment, individualized information guidance, attention to both cognitive and emotional aspects of expectations, and continued follow-up after RDN. Nurses may also help patients understand the potential benefits and limitations of RDN, particularly that medication reduction or discontinuation is not guaranteed. Such communication may support more realistic expectations, shared decision-making, and long-term self-management.

## Figures and Tables

**Table 1:** Sociodemographic Characteristics of Participants (*n* = 17).

Characteristic	*n* (%)
**Age group, years**	
Younger adults (18–44)	10 (58.8)
Middle-aged adults (45–59)	6 (35.3)
Older adults (≥60)	1 (5.9)
**Sex**	
Male	15 (88.2)
Female	2 (11.8)
**Education level**	
Primary school	2 (11.8)
Junior high school	3 (17.6)
Senior high or technical secondary school	3 (17.6)
College education or above	9 (52.9)
**Marital status**	
Married	14 (82.4)
Unmarried	2 (11.8)
Divorced	1 (5.9)
**Place of residence**	
Urban	12 (70.6)
Rural	5 (29.4)
**Type of medical insurance**	
Urban Employee Basic Medical Insurance	11 (64.7)
New Rural Cooperative Medical Scheme	5 (29.4)
Self-paying	1 (5.9)
**Family history of hypertension**	
Yes	16 (94.1)
No	1 (5.9)

**Note:** Data are presented as *n* (%). Age groups were defined for descriptive purposes according to the age distribution of the study sample.

**Table 2:** Individual Clinical Characteristics of Participants Before and After RDN.

Participant	Hypertension Duration, Years	Comorbidities, *n*	Baseline Office BP, mmHg	Medications Before RDN, *n*	Serum Creatinine, μmol/L	Post-RDN Office BP, mmHg	Medications After RDN, *n*	Interval from RDN to Interview, Months
P1	13	0	177/112	3	68.2	115/84	2	2
P2	7	0	170/120	4	88.8	133/75	2	1
P3	4	2	160/100	5	72.4	128/81	1	3
P4	3	1	180/112	4	87.9	153/98	3	2
P5	6	0	182/104	3	52.4	118/71	2	12
P6	7	0	170/99	4	72.0	131/81	2	3
P7	18	1	182/112	3	74.8	131/90	2	2
P8	11	1	200/105	5	84.3	134/89	4	1
P9	11	1	175/102	4	64.3	146/80	1	2
P10	7	0	173/104	3	216.8	129/85	2	2
P11	5	2	207/138	4	131.9	160/102	4	6
P12	5	2	190/110	4	80.8	125/82	2	5
P13	16	2	175/105	3	94.1	113/71	2	3
P14	18	3	172/110	4	78.5	139/93	3	6
P15	20	0	193/85	5	55.0	139/69	3	4
P16	20	2	164/92	3	98.6	131/85	3	2
P17	5	1	160/110	3	60.7	132/67	3	2

**Note:** BP, blood pressure; RDN, renal denervation. Office blood pressure is presented as systolic/diastolic values. Medication count refers to the number of antihypertensive drug classes taken before and after RDN.

## Data Availability

The data presented in this study are available on request from the corresponding author due to privacy and ethical restrictions. The qualitative interview transcripts contain potentially identifiable participant information and therefore cannot be made publicly available.

## Supplementary Materials

The following supporting information can be downloaded at https://www.mdpi.com/article/10.3390/healthcare14172786/s1. Supplementary Material S1. Code-to-Theme Pathway. Supplementary Material S2. Consolidated criteria for reporting qualitative research (COREQ): 32-item checklist. Ref. [13] is cited in the Supplementary Material.

## Abbreviations

The following abbreviations are used in this manuscript:

COREQ checklist	Consolidated Criteria for Reporting Qualitative Research checklist
RDN	Renal Denervation
SCT	Social Cognitive Theory
