# Supplementary material for: Patients’ Experiences of Outcome Expectations in Renal Denervation: A Qualitative Study
Source: Healthcare (Basel). 2026 Sep 1;14(17):2786. doi: 10.3390/healthcare14172786 (PMC13565244; doi:10.3390/healthcare14172786)
Supplement: Supplementary file 1 [file healthcare-14-02786-s001.zip › S1 Code-to-Theme Pathway.pdf]

Supplementary Material S1. Code-to-Theme Pathway

1. Analytic approach (summary)

Interviews were conducted in Mandarin Chinese and analysed using **inductive thematic analysis** in NVivo 20. Two researchers independently read the transcripts for familiarisation and conducted line-by-line **open coding**, identifying meaning units related to patients' expectations, perceptions, emotions, and support needs. Similar codes were compared, merged, and grouped into descriptive categories, producing an initial coding framework (Version 1.0) organised by illness- and treatment-related domains. Through iterative team review and constant comparison against the study aim, the organising axis of the analysis shifted from clinical/temporal domains to the **dynamics of outcome expectation itself**, yielding the final three-Theme structure (formation → regulation → readjustment). Disagreements in coding and interpretation were resolved by discussion until consensus, with a senior researcher arbitrating where needed. Successive versions of the coding framework were retained as an **audit trail** (summarised in Table S1-2).

Role of Social Cognitive Theory (SCT). Initial coding was conducted inductively, without applying predetermined SCT categories. SCT was consulted only after the inductive framework had been developed, and was used to **interpret** the relationships among the emerging categories (personal cognition and affect, prior experience, behaviour, and environmental influences, with outcome expectations as the central mechanism) — not to generate the codes or Themes. The final column of Table S1-1 therefore records SCT as an interpretive overlay applied post hoc.

Reflexivity. Interviews were conducted by a senior cardiovascular nurse trained in qualitative methods who was not involved in participants' RDN decision-making or postoperative management, reducing the likelihood that a treating relationship shaped participants' accounts. Quotations were translated into English for reporting.

2. Table S1-1. Code-to-Theme pathway

Theme 1 — Formation of Outcome Expectations

SubTheme 1.1 — Expectations Triggered by Prior Treatment Frustration

Representative raw data extract	Initial (open) codes	Category (2nd-level code)	Interpretive link to SCT (applied post hoc)
**P13:** "I first found out in 2009 — that's 16 years now."<br>**P14:** "For most people you just add one pill and it comes down. Not for me — adding drugs did nothing."<br>**P7:** "I went to Shanghai too, to Ruijin; the vascular tests at the Jiaotong University institute were all normal — nothing wrong."<br>**P5:** "It wasn't well controlled — systolic ~170, diastolic 98–100 — and very hard to keep stable."	– Long duration of hypertension<br>– Chronically poor BP control<br>– Repeated drug escalation still ineffective / conventional treatment "at its limit"<br>– Repeated help-seeking across hospitals<br>– Perceiving hypertension as "uncontrollable / refractory"	Prior experience of chronically uncontrolled blood pressure (disease-course & controllability perception)	**Enactive (mastery) experience** — an accumulated personal history of failed BP control acts as negative performance feedback, lowering confidence in conventional care and motivating the expectation that RDN might achieve what medication could not (personal-factor pathway).

SubTheme 1.2 — Expectations Strengthened by Risk Perception

Representative raw data extract	Initial (open) codes	Category (2nd-level code)	Interpretive link to SCT (applied post hoc)
High threat<br>**P1:** "The hospital made it sound terrifying — that I could suddenly drop dead: cardiac arrest, a cardiovascular blockage."<br>**P17:** "My father had a cerebral infarction; a vessel burst, half his body was paralyzed, and he died at 52."<br>**P14:** "My father, grandfather and aunt all had strokes … my aunt is now paralyzed."<br>Low threat<br>**P9:** "The doctor mentioned it, but I don't really care — as long as it isn't affecting me now."	– Fear of sudden death / cardio-cerebrovascular complications<br>– Threat perception amplified by family members' experiences & family history<br>– Threat cognition partly from the physician's warning<br>– (low) "If I don't think about it, it's fine"<br>– (low) Indifferent / threat-minimising attitude	Fear versus indifference toward long-term complication risk — the two poles of threat perception (consequence cognition & threat perception)	**Cognitive appraisal of risk (personal factor), amplified by vicarious/environmental input** — perceived severity, heightened by observing adverse outcomes in relatives, raises the perceived value of RDN and strengthens outcome expectations; threat-minimising appraisal yields reserved expectations.

**P16:** "I've basically never thought about it … I'm an easy-going person."			

SubTheme 1.3 — Treatment Expectations Driven by Medication Burden

Representative raw data extract	Initial (open) codes	Category (2nd-level code)	Interpretive link to SCT (applied post hoc)
**P15:** "I'm very sensitive — one drug made my feet swell, another gave me a cough. Neither this nor that works."<br>**P3:** "It was the eczema and insomnia I couldn't bear — it healed and came back … agonizing."<br>**P16:** "Before, I just took drugs, and when it wouldn't come down I asked the doctor to switch them."	– Severe drug side-effects affecting daily life<br>– Frustration from frequent medication changes<br>– Poor drug control / loss of confidence in medication<br>– Long-term / lifelong medication is burdensome<br>– Hope to reduce or stop medication	Limitations of pharmacotherapy and the burden of side-effects (medication experience & controllability perception)	**Behaviour–outcome contingency from direct experience** — the lived experience that daily medication is burdensome and only partly effective (a personal/behavioural history) drives the expectation that an alternative route will lighten the medication burden.

SubTheme 1.4 — Expectations Shaped by Social Information

Representative raw data extract	Initial (open) codes	Category (2nd-level code)	Interpretive link to SCT (applied post hoc)
**P5:** "I was terrified that a cerebral hemorrhage would affect my whole family — my two children, my husband … my husband supported the decision."<br>**P3:** "Before me, someone had it done here — he was on four drugs, and afterwards only one."<br>**P16:** "The doctor said the procedure might bring my blood pressure down. With opinions from several sides, I went ahead."<br>**P7:** "I read magazines and often came across it — I'd been following this technique a long time."	– Family responsibility motivating active treatment / avoiding becoming a burden<br>– Family support for the decision<br>– Peers'/friends' success stories strengthening confidence<br>– Physician recommendation & authority<br>– Information via social networks / short-video platforms / magazines	Influence of multiple external factors on the decision and expectations (core motives for choosing RDN + sources of outcome expectations)	**Environmental factors + observational (vicarious) learning** — peers' visible success (modeling), physician recommendation (verbal persuasion/authority) and family-role expectations feed into outcome expectations; the clearest environment→cognition direction in SCT's reciprocal determinism.

Theme 2 — Regulation of Outcome Expectations

SubTheme 2.1 — Cognitive Trade-offs Triggered by Uncertainty Regarding Surgical Efficacy

Representative raw data extract	Initial (open) codes	Category (2nd-level code)	Interpretive link to SCT (applied post hoc)
**P3:** "Even my attending physician didn't know; I later learned the ablation sites are chosen somewhat by luck — a bit of 'blind ablation'."<br>**P6:** "Before procedure I didn't expect any particular effect — it's a new technique, so nothing is certain."<br>**P17:** "The doctor said it varies from person to person — for some a bit less medication, for others much the same."<br>**P7:** "It's impossible to just stop medication because the procedure is done — it varies from person to person."	– Recognising that efficacy varies between individuals<br>– Viewing it as a new technique with uncertain effect<br>– Awareness of mechanism limits ("blind ablation")<br>– Learning of uncertainty through preoperative doctor–patient communication<br>– procedure is "not 100%," a matter of probability	Cognition of surgical-efficacy uncertainty (psychological anticipation of a non-ideal outcome — cognitive dimension)	**Cognitive processing of efficacy information** — appraisal of uncertainty, drawn from physician communication and understanding of the mechanism, tempers the outcome expectation; the cognitive dimension of an outcome expectation being actively adjusted.

SubTheme 2.2 — Establishment of Defensive Psychological Expectations

Representative raw data extract	Initial (open) codes	Category (2nd-level code)	Interpretive link to SCT (applied post hoc)
**P5:** "I'd prepared myself — if it didn't work, that's fine; at worst it stays the same as before (160 or 170)."<br>**P6:** "Could the worst be worse than now? At worst, no effect — a drop of three to five points — I'd accept that; I had to try."<br>**P14:** "Even if there's no effect, I still want to try — you have to walk every possible path."<br>**P8:** "I treated it as one more cut — give it a try, maybe it works; the worst is simply no effect."	– Presetting "the worst is no effect / same as before"<br>– Not over-expecting; lowering expectations to relieve pressure<br>– Exploratory, tentative stance ("give it a try anyway")<br>– As long as it does no harm, no effect is acceptable	Establishing defensive psychological expectations (psychological anticipation of a non-ideal outcome — affective/self-protective dimension)	**Self-regulatory / self-protective expectation setting** — pre-committing to a "worst case = no change" floor is an anticipatory affect-regulation strategy that pre-empts disappointment; the affective dimension of regulating outcome expectations.

Theme 3 — Readjustment of Outcome Expectations

SubTheme 3.1 — Validation of Expectations Based on Outcome Feedback

Representative raw data extract	Initial (open) codes	Category (2nd-level code)	Interpretive link to SCT (applied post hoc)
**P5:** "After the procedure my systolic came down and became stable … fairly ideal. I feel it was well worth it."<br>**P16:** "Quite useful — now medication controls my blood pressure, whereas before it couldn't … it basically met my expectations."<br>**P7:** "Worth it — at the very least I can take less medication."<br>**P12:** "Quite worth it, in line with what I expected."<br>**P17:** "Judging from the data I'm satisfied — it holds around 130–140."	– Effective, stable postoperative BP control<br>– Reduced medication requirement<br>– Surgical effect met inner expectation<br>– Considering the procedure worthwhile<br>– Disappearance of symptoms (e.g., dizziness)	Fulfilment of postoperative outcome expectations — expectation validation (surgical outcomes & feelings, positive feedback)	**Outcome feedback confirming expectations** — positive postoperative results (environmental/behavioural feedback) validate and reinforce the prior outcome expectation; the reinforcement loop within reciprocal determinism.

SubTheme 3.2 — Emotional Experiences Triggered by Expectation Discrepancy

Representative raw data extract	Initial (open) codes	Category (2nd-level code)	Interpretive link to SCT (applied post hoc)
**P15:** "After I came home, my blood pressure suddenly shot up again … I've lost all confidence … there's definitely some disappointment."<br>**P2:** "It's not quite [what I imagined] — I still have to keep taking medication."<br>**P14:** "The first morning it was a bit better, now it's back to before — it feels as if the procedure was for nothing … and that night the pain was unbearable."<br>**P4:** "There's a gap — some effect, just not as good as I'd expected."<br>**P13:** "Not fully achieved — some effect, no more 170/180, but not obvious."	– Surgical effect fell short of expectation<br>– Postoperative BP rebound → disappointment / doubt about success<br>– Surgical pain exceeding expectation<br>– A gap in effect and a sense of loss<br>– Some effect but still a gap	The felt gap between expected and actual outcome — negative emotion (surgical outcomes & feelings, expectation discrepancy)	**Expectation–outcome discrepancy generating affect** — the mismatch between anticipated and actual outcomes (including pain exceeding expectation) produces disappointment; the affective consequence of a violated outcome expectation.

SubTheme 3.3 — Psychological Adjustment Driven by Unfavorable Outcomes

Representative raw data extract	Initial (open) codes	Category (2nd-level code)	Interpretive link to SCT (applied post hoc)
**P14:** "But what can be done? It was my own decision; I can't blame the doctors."<br>**P15:** "There's no other way — whether I accept it or not, I have to accept it."<br>**P3:** "Later I came to terms with it and grew calm — this kind of thing may just be fated."	– Resigned acceptance of the gap<br>– Attributing it to one's own decision / not blaming doctors<br>– Attributing it to fate / the limits of medicine<br>– Moving from disappointment to calm acceptance	Psychological adjustment and acceptance after an unsatisfactory outcome (surgical outcomes & feelings, psychological adjustment)	**Self-regulation via cognitive reappraisal** — where superior alternatives are limited, patients recalibrate expectations through acceptance and reinterpretation; a self-regulatory adjustment of cognition and affect to environmental reality.

SubTheme 3.4 — Behavioral Adjustment Guided by Support Needs

Representative raw data extract	Initial (open) codes	Category (2nd-level code)	Interpretive link to SCT (applied post hoc)
**P15:** "We aren't good with smartphones — I get my daughter to handle registration. Other help, it's hard for them to give."<br>**P6:** "What's the safe BP range for exercise — for swimming? For intimacy, I worry about BP rising, so I really need a doctor's guidance. Online info isn't professional."<br>**P7:** "If any new drug or technique comes along … let us know … a regimen without long-term medication, or an injection."<br>**P9:** "If there's a particularly effective new antihypertensive, just recommend it and I'll try it."	– Viewing BP management as a personal responsibility; reluctance to trouble family<br>– Family unable to provide substantive help<br>– Need for professional guidance (activity / intimacy safety thresholds)<br>– Expecting long-term clinician follow-up & feedback<br>– Wanting the latest medication/technique information	Reliance on professional support and avoidance of family reliance (support needs — family & healthcare providers)	**Environment–behaviour interaction in self-management** — help-seeking directed toward professional support and away from family reflects the environmental component of the triad and self-efficacy considerations in sustaining long-term health behaviour.

3. Table S1-2. Audit trail — evolution of the analytic/thematic structure

Stage	Organising principle	Theme structure	What changed, and why
**v1.0** (initial NVivo framework)	Descriptive, organised by **illness/treatment domain**	① Formation of illness experience & representation; ② Construction of outcome expectations in surgical decision-making; ③ Behavioural preparation & self-efficacy; ④ Support needs; ⑤ Other	Product of inductive open + axial coding. Categories describe content areas, **not** SCT constructs — the framework is data-derived.
**v2.0**	First reorganisation toward a **process** reading	① Illness experience & risk perception jointly driving the RDN decision; ② Formation drivers of surgical outcome expectations; ③ Rational trade-offs & psychological adjustment in expectation-management decisions; ④ Expectation discrepancy & coping in the postoperative experience	Re-examining v1.0 against the study aim, the team saw the same material as a temporal sequence centred on outcome expectations and began regrouping categories around that process.
**v3.0 / v4.0**	Refined process structure (labels tightened; v4.0 = wording stabilisation of v3.0)	① Long-term treatment-frustration experience & risk perception; ② Formation sources of surgical outcome expectations; ③ Rational trade-offs & psychological-expectation management; ④ Fulfilment vs discrepancy adjustment of postoperative outcome expectations	Labels made parallel; the postoperative Theme separated more clearly into satisfaction / discrepancy / adjustment / support.
**v5.0** (final, Chinese)	The expectation **process named directly**	① Formation of outcome expectations (4 subThemes); ② Regulation of outcome expectations (2); ③ Readjustment of outcome expectations (4)	Four Themes consolidated into three; overlap between "formation sources" and "risk perception" removed; the cognitive vs defensive facets of regulation separated → 10

			bounded subThemes.
**Final** (manuscript, English)	—	Direct English rendering of v5.0 (3 Themes, 10 subThemes)	SubTheme wording finalised for reporting.

Note. Social Cognitive Theory was consulted only **after** the initial inductive framework (v1.0) had been developed; it informed the interpretation of relationships among categories and is not reflected in the descriptive, domain-based organisation of v1.0 — evidence that the Themes are grounded in the data rather than deduced from the theory.
