# Supplementary material for: Patients’ Experiences of Outcome Expectations in Renal Denervation: A Qualitative Study
Source: Healthcare (Basel). 2026 Sep 1;14(17):2786. doi: 10.3390/healthcare14172786 (PMC13565244; doi:10.3390/healthcare14172786)
Supplement: Supplementary file 1 [file healthcare-14-02786-s001.zip › S2 COREQ Checklist.pdf]

Consolidated criteria for reporting qualitative studies (COREQ): 32-item checklist

Developed from:

Tong A, Sainsbury P, Craig J. Consolidated criteria for reporting qualitative research (COREQ): a 32-item checklist for interviews and focus groups. *International Journal for Quality in Health Care*. 2007. Volume 19, Number 6: pp. 349 – 357

MANUSCRIPT TITLE: Patients' Experiences of Outcome Expectations in Renal Denervation: A Qualitative Study

No. Item	Guide questions/description	Reported in manuscript section / location
**Domain 1: Research team and reflexivity**		
Personal Characteristics		
1. Inter viewer/facilitator	Which author/s conducted the interview or focus group?	Section 2.2.2 Data Collection Methods: Supplementary Material S2: The interviews were conducted by D.H., a senior cardiovascular nurse trained in qualitative research.
2. Credentials	What were the researcher's credentials? (E.g. PhD, MD)	Supplementary Material S2 (this checklist): the interviewer was a master's student.
3. Occupation	What was their occupation at the time of the study?	Section 2.2.2 Data Collection Methods: senior cardiovascular nurse.
4. Gender	Was the researcher male or female?	Supplementary Material S2 (this checklist): the interviewer was female.
5. Experience and training	What experience or training did the researcher have?	Section 2.2.2 Data Collection Methods: The interviewer was trained in qualitative research methods.
Relationship with participants		
6. Relationship established	*Was a relationship established prior to study commencement?* .	Section 2.2.2 Data Collection Methods: interviews were conducted after discharge, and the interviewer was not directly involved in participants' RDN decision-making or postoperative treatment management.
7. Participant knowledge of the interviewer	*What did the participants know about the researcher? (e.g. personal goals, reasons for doing the research).*	Section 2.2.2 Data Collection Methods: study purpose, content, and significance were explained before informed consent was obtained.
8. Interviewer characteristics	*What characteristics were reported about the interviewer/facilitator? (e.g. Bias, assumptions, reasons and interests in the research topic)*	Sections 2.2.2 and 4.4: The interviewer was a senior cardiovascular nurse trained in qualitative research and was not directly involved in participants' clinical decision-making or postoperative management. The manuscript acknowledges possible interviewer or question-framing bias.
**Domain 2: Study design**		
Theoretical framework		
9. Methodological orientation and Theory	*What methodological orientation was stated to underpin the study? (e.g. grounded theory, discourse analysis, ethnography, phenomenology, content analysis).*	Abstract and Section 2.2.3 Data Analysis: qualitative descriptive design; inductive thematic analysis. Introduction and Section 2.2.3: Social Cognitive Theory used as an interpretive framework after preliminary codes/categories were developed.
Participant selection		
10. Sampling	*How were participants selected? (e.g. purposive, convenience, consecutive, snowball)*	Section 2.1 Participants: purposive sampling incorporating maximum variation sampling.
11. Method of approach	*How were participants approached? (e.g. face-to-face, telephone, mail, email)*	Section 2.1 Participants and Section 2.2.2 Data Collection Methods: Eligible patients were identified and recommended by the clinical team according to the study criteria. The researcher then contacted potential participants, explained the study, and invited them to participate. Formal interviews were subsequently conducted by telephone after discharge.

Information Classification: General

No. Item	Guide questions/description	Reported in manuscript section / location
12. Sample size	*How many participants were in the study?*	Section 2.1 Participants and Figure 1: Nineteen patients consented to participate; two participated in pilot interviews and were excluded from the final analysis, leaving 17 participants in the final qualitative sample.
13. Non-participation	*How many people refused to participate or dropped out? Reasons?*	Section 2.1 Participants and Figure 1: Of 21 eligible patients invited to participate, two declined because of time constraints. Nineteen consented, of whom two participated in pilot interviews and were excluded from the final qualitative analysis. The remaining 17 participants completed the study, and none withdrew.
Setting		
14. Setting of data collection	*Where was the data collected? (e.g. home, clinic, workplace)*	Section 2.2.2 Data Collection Methods: semi-structured telephone interviews were conducted after discharge, at varying intervals following RDN.
15. Presence of non-participants	*Was anyone else present besides the participants and researchers?*	Supplementary Material S2 (this checklist): interviews were conducted by telephone, and the presence of non-participants at the participants' location was not recorded.
16. Description of sample	*What are the important characteristics of the sample? (e.g. demographic data, date)*	Section 2.1 Participants and Tables 1 and 2: Table 1 presents sociodemographic characteristics, including age, sex, education level, marital status, place of residence, medical insurance, and family history of hypertension. Table 2 presents relevant clinical characteristics, including hypertension duration, comorbidities, blood pressure, medication use, renal function, and the interval from RDN to interview.
Data collection		
17. Interview guide	*Were questions, prompts, guides provided by the authors? Was it pilot tested?*	Section 2.2.1 Development of Interview Guides: interview guide was developed from study aims, literature, and SCT; refined by team discussion; pilot-tested with two patients; final questions are listed.
18. Repeat interviews	*Were repeat interviews carried out? If yes, how many?*	Supplementary Material S2 (this checklist): no repeat interviews were conducted.
19. Audio/visual recording	*Did the research use audio or visual recording to collect the data?*	Section 2.2.2 Data Collection Methods: with participants' permission, each interview was audio-recorded in full.
20. Field notes	*Were field notes made during and/or after the interview or focus group?*	Section 2.2.3 Data Analysis: audible paralinguistic cues, including pauses, tone of voice, and emotional expressions, were documented during transcription. Separate field notes are not otherwise reported.
21. Duration	*What was the duration of the inter views or focus group?*	Section 2.2.2 Data Collection Methods: each interview lasted 30-40 minutes.
22. Data saturation	Was data saturation discussed?	Section 2.1 Participants: thematic saturation defined as no new subthemes or themes during concurrent analysis; assessed by two researchers; two further interviews confirmed saturation.
23. Transcripts returned	*Were transcripts returned to participants for comment and/or correction?*	Section 2.2.3 Data Analysis: Transcripts were returned to participants for verification. Fifteen participants confirmed their accuracy, while the remaining two participants did not respond after two contact attempts. Only minor wording clarifications were made.
**Domain 3: analysis and findings**		
Data analysis		
24. Number of data coders	How many data coders coded the data?	Section 2.2.3 Data Analysis: two researchers independently read transcripts and conducted open coding.
25. Description of the coding tree	*Did authors provide a description of the coding tree?*	Section 2.2.3 Data Analysis and Supplementary Material S1 Code-to-theme pathway: meaningful units, open codes, preliminary categories, subthemes, and themes are described; S1 presents the code-to-theme pathway.
26. Derivation of themes	*Were themes identified in advance or derived from the data?*	Section 2.2.3 Data Analysis: themes were derived inductively from the data; initial coding did not apply predetermined SCT categories; SCT was consulted after preliminary codes/categories were developed.
27. Software	*What software, if applicable, was used to manage the data?*	Section 2.2.3 Data Analysis: NVivo 20 software was used for data management and coding.
28. Participant	*Did participants provide feedback on the*	Section 2.2.3 Data Analysis: Interview transcripts were returned to

Information Classification: General

No. Item	Guide questions/description	Reported in manuscript section / location
checking	*findings?*	participants for verification. Fifteen participants confirmed the accuracy of the transcripts, while two did not respond after two contact attempts. Only minor wording clarifications were made, and no codes or themes were modified.
Reporting		
29. Quotations presented	*Were participant quotations presented to illustrate the themes/findings? Was each quotation identified? (e.g. participant number)*	Section 3 Results: representative quotations are presented throughout Sections 3.1-3.3 and identified by participant number (e.g., P1-P17).
30. Data and findings consistent	*Was there consistency between the data presented and the findings?*	Section 3 Results: each theme and subtheme is supported by participant quotations. Supplementary Material S1 Code-to-theme pathway provides the audit trail linking raw data, codes, categories, subthemes, and themes.
31. Clarity of major themes	*Were major themes clearly presented in the findings?*	Section 3 Results: three major themes are clearly presented: formation of outcome expectations, regulation of outcome expectations, and readjustment of outcome expectations.
32. Clarity of minor themes	Is there a description of diverse cases or discussion of minor themes?	Section 3 Results: ten subthemes are presented under the three major themes; diverse cases are described, including high versus low risk perception and satisfied versus discrepant postoperative experiences.
